# Effects of Lianhuaqingwen Capsules in adults with mild-to-moderate coronavirus disease 2019: an international, multicenter, double-blind, randomized controlled trial

**DOI:** 10.1186/s12985-023-02144-6

**Published:** 2023-11-28

**Authors:** Jin-ping Zheng, Yun Ling, Liang-shuang Jiang, Piroon Mootsikapun, Hong-zhou Lu, Methee Chayakulkeeree, Li-xiu Zhang, Pureepat Arttawejkul, Feng-yu Hu, Thi Ngoc Lan Truong, Roxan A. Perez, Xing Gu, Hui-min Sun, Jian-jie Jiang, Ren-jie Liu, Zhen Ding, Yang-qing Zhan, Zi-feng Yang, Wei-jie Guan, Nan-shan Zhong

**Affiliations:** 1 State Key Laboratory of Respiratory Disease, National Clinical Research Center for Respiratory Disease, Guangzhou Institute for Respiratory Health, The First Affiliated Hospital of Guangzhou Medical University, 28 Qiaozhong Road Middle, Guangzhou, Guangdong China; 2 Shanghai Public Health Clinical Center, Shanghai, China; 3 Public Health Clinical Center of Chengdu, Chengdu, China; 4 Srinagarind Hospital, Khon Kaen, Thailand; 5 The Third People’s Hospital of Shenzhen, Shenzhen, China; 6 Siriraj Hospital, Khon Kaen, Thailand; 7 The Affiliated Hospital of Changchun University of TCM, Changchun, China; 8 Burapha University Hospital, Khon Kaen, Thailand; 9 Guangzhou Eighth People’s Hospital, Guangzhou Medical University, Guangzhou, China; 10 Traditional Medicine Institute of Ho Chi Minh City, Ho Chi Minh City, Vietnam; 11 Dr. Jose N. Rodriguez Memorial Hospital and Sanitarium, Caloocan, The Philippines; 12 Xi’an Chest Hospital, Xi’an, China; 13 Tangshan Hospital of Traditional Chinese Medicine, Tangshan, China; 14 Kunming Third People’s Hospital, Kunming, China; 15 First People’s Hospital of Zhengzhou City, Zhengzhou, China; 16 Hefei Binhu Hospital, Hefei, China

**Keywords:** Coronavirus disease 2019, Omicron, Lianhuaqingwen Capsule, Symptom resolution, Inflammation

## Abstract

**Background:**

In a randomized trial, Lianhuaqingwen (LHQW) capsule was effective for accelerating symptom recovery among patients with coronavirus disease 2019 (COVID-19). However, the lack of blinding and limited sample sizes decreased the level of clinical evidence.

**Objectives:**

To evaluate the efficacy and safety of LHQW capsule in adults with mild-to-moderate COVID-19.

**Methods:**

We conducted a double-blind randomized controlled trial in adults with mild-to-moderate COVID-19 (17 sites from China, Thailand, Philippine and Vietnam). Patients received standard-of-care alone or plus LHQW capsules (4 capsules, thrice daily) for 14 days. The primary endpoint was the median time to sustained clinical improvement or resolution of nine major symptoms.

**Results:**

The full-analysis set consisted of 410 patients in LHQW capsules and 405 in placebo group. LHQW significantly shortened the primary endpoint in the full-analysis set (4.0 vs. 6.7 days, hazards ratio: 1.63, 95% confidence interval: 1.39-1.90). LHQW capsules shortened the median time to sustained clinical improvement or resolution of stuffy or runny nose (2.8 vs. 3.7 days), sore throat (2.0 vs. 2.6 days), cough (3.2 vs. 4.9 days), feeling hot or feverish (1.0 vs. 1.3 days), low energy or tiredness (1.3 vs. 1.9 days), and myalgia (1.5 vs. 2.0 days). The duration to sustained clinical improvement or resolution of shortness of breath, headache, and chills or shivering did not differ significantly between the two groups. Safety was comparable between the two groups. No serious adverse events were reported.

**Interpretation:**

LHQW capsules promote recovery of mild-to-moderate COVID-19 via accelerating symptom resolution and were well tolerated.

*Trial registration* ChiCTR2200056727.

**Supplementary Information:**

The online version contains supplementary material available at 10.1186/s12985-023-02144-6.

## Introduction

Severe acute respiratory syndrome coronavirus 2 (SARS-CoV-2), the culprit of coronavirus disease 2019 (COVID-19), is continuously evolving globally [1]. The Omicron variant has a substantially increased transmissibility and decreased pathogenicity in lower airways compared with the ancestral strain and existing variants [2, 3]. Apart from fever and fatigue, the Omicron variant elicits respiratory symptoms (e.g. cough, shortness of breath) that markedly reduced quality-of-life and caused a substantial morbidity and mortality among the vulnerable populations.

Several therapeutic approaches have been developed to reduce the symptom burden and the probability of progression into severe or critical illness. These mainly include antivirals (e.g. nirmatrelvir-ritonavir, monulpiravir, VV116) [4–6], monoclonal antibodies (e.g. tixagavimab-cilgavimab, sotrovimab, tocilizumab) [7–9] and anti-inflammatory drugs (e.g. baricitinib, tofacitinib) [10, 11]. For most patients with mild-to-moderate diseases managed at home or communities, there remains a paucity of less costly marketed medications for symptomatic amelioration.

Several traditional Chinese medicine formulae have been marketed and endorsed for clinical application by the *Protocol of the Diagnosis and Treatment of Coronavirus Disease 2019* (10th version, Ministry of Health, China) [12]. Lianhuaqingwen (LHQW) capsules reportedly inhibited SARS-CoV-2 replication and conferred anti-inflammatory activity *in vitro*-suppression of pro-inflammatory cytokines (tumor necrosis factor-α, interleukin-6, C-C motif ligand-2/Macrophage chemotaxis protein-1 and CXC motif chemokine ligand-10/Inducible protein-10) production at the mRNA levels [13]. In a case-series observational study, LHQW markedly ameliorated the cardinal symptoms and accelerated recovery of COVID-19 [14]. In a randomized controlled trial among 284 patients infected with the ancestral strain, LHQW (four capsules, thrice daily) for 14 days yielded significantly higher rate of clinical recovery (91.5% vs. 82.4%, *P*=0.022), accelerated symptom recovery (median: 7 vs. 10 days, *P*<0.001), and a higher rate of chest computed tomography (CT) improvement (83.8% vs. 64.1%, *P*<0.001) and clinical cure (78.9% vs. 66.2%, *P=*0.017) compared with control group [15]. Because of the rapidly evolving outbreak, that multicenter trial suffered from major limitations such as no stringent randomization and the inclusion of Chinese patients only.

We conducted the randomized, double-blind, international, multicenter, investigator-initiated clinical trial to investigate the efficacy and safety of Lianhuaqingwen capsules compared to placebo and combined with standard-of-care in adult patients with mild-to-moderate COVID-19 (FLOSAN study). We hypothesized that LHQW capsules would accelerate symptom recovery among adults with mild-to-moderate COVID-19.

## Methods

### Study design and patients

The FLOSAN trial recruited patients with mild-to-moderate COVID-19 from 12 hospitals in China, 3 hospitals in Thailand, and one hospital each in Vietnam and the Philippines between February and December 2022 (Additional file 1: Table S1). The full version of study protocol has been published recently (See details in Online Supplement) [16]. Briefly, eligible patients were aged 18-70 years, had mild-to-moderate COVID-19 (according to *World Health Organization* criterion) [17], tested positive to either rapid antigen test (RAT) or nucleic acid amplification test (NAAT), had an interval between symptom onset and screening of within 4 days, and had at least three major symptoms (stuffy or runny nose, sore throat, cough, shortness of breath, low energy or tiredness, myalgia, headache, chills or shivering, feeling hot or feverish) occurring within 12 hours prior to screening. We excluded patients who had: (1) known co-morbidities of other infections; (2) poorly controlled systemic diseases; (3) alcohol or drug abuse within one year; (4) participated in other trials within one month; (5) become pregnant, breastfeeding or within two weeks of delivery. The FLOSAN trial was conducted in accordance with the *Declarations of Helsinki*. Ethics approval has been obtained the ethics committee of each participating site, based on *Good Clinical Practice*. All patients signed written informed consent.

**Table 1:** Baseline demographic and clinical characteristics of the full-analysis set

	LHQW group (N = 410)	Placebo group (N = 405)	Total (N = 815)
Age (yrs), Mean ± SD	37.8 ± 14.5	36.3 ± 13.1	37.1 ± 13.8
Females, n (%)	196 (47.8)	216 (53.3)	412 (50.6)
Height (cm), Mean ± SD	166.3 ± 9.0	165.7 ± 8.4	166.0 ± 8.7
BMI (kg/m^2^), Mean ± SD	23.6 ± 3.7	23.2 ± 3.7	23.4 ± 3.7
Nationality (region)			
China, n (%)	249 (60.7)	249 (61.5)	498 (61.1)
Hong Kong (China), n (%)	3 (0.7)	1 (0.2)	4 (0.5)
Vietnam, n (%)	22 (5.4)	21 (5.2)	43 (5.3)
Thailand, n (%)	117 (28.5)	113 (27.9)	230 (28.2)
Philippine, n (%)	10 (2.4)	11 (2.7)	21 (2.6)
Others, n (%)	9 (2.2)	10 (2.5)	19 (2.3)
Local citizens, n (%)	368 (89.8)	357 (88.1)	725 (89.0)
Ethnicity	410	405	815
Yellow, n (%)	400 (97.6)	393 (97.0)	793 (97.3)
Brown, n (%)	10 (2.4)	12 (3.0)	22 (2.7)
COVID-19 vaccination			
Primary-series, n (%)	370 (90.2)	357 (88.1)	727 (89.2)
Incomplete, n (%)	18 (4.4)	27 (6.7)	45 (5.5)
Not vaccinated, n (%)	22 (5.4)	21 (5.2)	43 (5.3)
Receipt of COVID-19 vaccines			
Inactivated vaccines	262 (67.5)	241 (62.8)	503 (65.2)
Adenovirus vaccines	55 (14.2)	57 (14.8)	112 (14.5)
mRNA vaccines	133 (34.3)	148 (38.5)	281 (36.4)
Recombinant vaccines	5 (1.3)	7 (1.8)	12 (1.6)
Missing information	23 (5.9)	25 (6.5)	48 (6.2)
Duration of vaccination to enrollment (d)	194.8 ± 103.7	189.8 ± 99.9	192.3 ± 101.8
Symptoms			
Cough, n (%)	353 (86.1)	352 (86.9)	705 (86.5)
Sore throat, n (%)	330 (80.5)	342 (84.4)	672 (82.5)
Stuffy or runny nose, n (%)	313 (76.3)	319 (78.8)	632 (77.5)
Low energy or tiredness, n (%)	247 (60.2)	253 (62.5)	500 (61.3)
Myalgia, n (%)	221 (53.9)	220 (54.3)	441 (54.1)
Comorbidities			
Hypertension,n%	34 (8.3)	23 (5.7)	57 (7.0)
Diabetes,n%	9 (2.2)	6 (1.5)	15 (1.8)
Fatty liver disease,n%	8 (2.0)	4 (1.0)	12 (1.5)
Dyslipidemia,n%	7 (1.7)	5 (1.2)	12 (1.5)
Allrgic rhinitis,n%	2 (0.5)	7 (1.7)	9 (1.1)
Charlson's comorbidity score	0.70 ± 1.08	0.59 ± 0.97	0.64 ± 1.03
Charlson's comorbidity score ≤ 2, n(%)	377 (92.0)	380 (93.8)	757 (92.9)
Charlson's comorbidity score > 2, n(%)	33 (8.0)	25 (6.2)	58 (7.1)

### Randomization and masking

We randomly assigned patients (1:1) to receive treatment with LHQW or matching placebo (manufactured by Shijiazhuang Yiling Pharmaceutical Co. Ltd., Shijiazhuang, China) based on the randomization numbers generated with the SAS package (SAS Inc., Cary, USA). The block size was 4 with no stratification. With competitive recruitment scheme, the sub-site investigators allocated patients in an ascending order. The study medications had an identical color, odor and appearance, except that the placebo did not contain any active ingredient of LHQW. Patients, the study investigators and other staff were masked to treatment allocation until database lock.

### Procedures

After randomization, patients took LHQW (4 capsules [0.35g/capsule], thrice daily) or matching placebo for 14 consecutive days following hospitalization in designated hospitals (in mainland China) and out-patient recruitment (in the Phillipines, Thailand and Viet Nam). Both groups received standard-of-care consisting of antipyretics, analgesic drugs, nutrition supplementation and fluid replacement. Acetaminophen, the non-steroidal anti-inflammatory drug, could be applied for ameliorating fever if the temperature reached 38.5 degrees or higher. Antivirals or medications with core components of LHQW were prohibited. Sites could follow local guidelines and protocols in their countries and regions. Patients attended four in-hospital (in mainland China) or out-patient (in Thailand, Vietnam and the Philippines) visits (days 3, 7, 10 and an end-of-study visit, typically scheduled at day 14). Patients who prematurely discontinued treatment due to accelerated symptom recovery or other reasons could attend all planned visits. During the study, patients were requested to fill out the diary card twice daily to evaluate the changes in symptoms.

### Outcomes

The primary endpoint was evaluated at day 14 - the median time to sustained clinical improvement or resolution of the nine above-mentioned major symptoms, rated as being less than or equal to mild (scored 1 or 0) and remained stable for >24 hours (see Supplementary File for the symptom diary cards).

Pre-specified secondary endpoints included the proportion of patients with sustained improvement or resolution of nine major symptoms at day 14, the median time to sustained improvement or resolution of each of these individual symptoms, the median time to onset of antipyretic effect and return to normal temperature (axillary temperature ≤ 37.0°C or oral temperature ≤ 37.3°C for >24 hours), the median time to sustained improvement or resolution of gastrointestinal symptoms, anosmia and ageusia, the proportion of patients with sustained improvement or resolution of all symptoms, the time to negative conversion of NAAT findings, and the rate of NAAT negative conversion (days 0, 7, 10, 14), the proportion of patients with major improvement in chest imaging, the incidence of COVID-19-related severe/critical disease, COVID-19-related and all-cause mortality within day 14. A designated experienced radiologist (blinded to study allocation) reviewed chest X-ray or computed tomography (CT) images and rated the outcomes. An improvement in chest radiology denoted a decreased area of infiltration, a decreased area of any radiologic abnormality, or decreased density of ground-glass opacity or nodules [15].

Safety endpoints were evaluated from the first dosing to the end of follow-up, including vital signs, physical examination, major changes in laboratory test, abnormal twelve-lead electrocardiogram findings, and the adverse event (AE) and serious adverse event (SAE). See *Online Supplement* for details.

### Statistical analysis

Assuming that the median time to sustained improvement or resolution was 12 days in control group and 9 days in LHQW group, 652 patients would be randomized to LHQW or placebo group (1:1) with a 95% power with a two-sided significance of 0.05 according to PASS software. In practice, patients were enrolled while taking into account RAT findings, and 344 patients per group would be needed when assuming that 95% of patients with positive RAT findings would yield positive NAAT findings. Recruitment of 860 patients would be needed while considering a 20% dropout rate.

We conducted statistical analyses with SAS 9.4 software (SAS Institute, Cary, North Carolina). All patients who had been randomized and taken at least one dose of study medication and had a confirmed diagnosis of COVID-19 based on NAAT were included in the full-analysis set. Patients who fully complied with the protocol (adherence: 80% or greater) were included in per-protocol set. We prioritized data presentation of the full-analysis set. The primary endpoint was analyzed by using the Log-rank test and displayed with Kaplan-Meier curve. The time to events was presented as the median duration and 95% confidence interval (95%CI). The hazards ratio (HR) of clinical events was demonstrated. We analyzed the following endpoints with chi-square test or Fisher’s exact probability model, including the proportion of patients with alleviation of symptoms, reduction in viral shedding (censored at day 14), major improvement in radiology, severe and critical diseases, death and all-cause death. We also analyzed the median time to sustained alleviation of single symptom, the alleviation of fever, digestive symptoms, ageusia or anosmia and all clinical symptoms, and the duration of viral shedding with the same analytical strategy with the primary endpoint. We conducted *post-hoc* subgroup analysis of the primary endpoint according to the strata of nationality, sex, age, vaccination status, concomitant antiviral drugs or other Traditional Chinese Medicine compounds, and the duration of symptom onset.

The FLOSAN trial was registered with Chinese Clinical Trial Registry (No.: ChiCTR2200056727). The CONSORT checklist can be found in the supplemental file.

### Role of funding source

The sponsor participated in the study design along with the principal investigators, study medication provision and data collection. An independent third party participated in data analysis. The first and corresponding authors had full access to the data and the corresponding author had the final decision to submit the manuscript for publication.

## Results

**Fig. 1:**
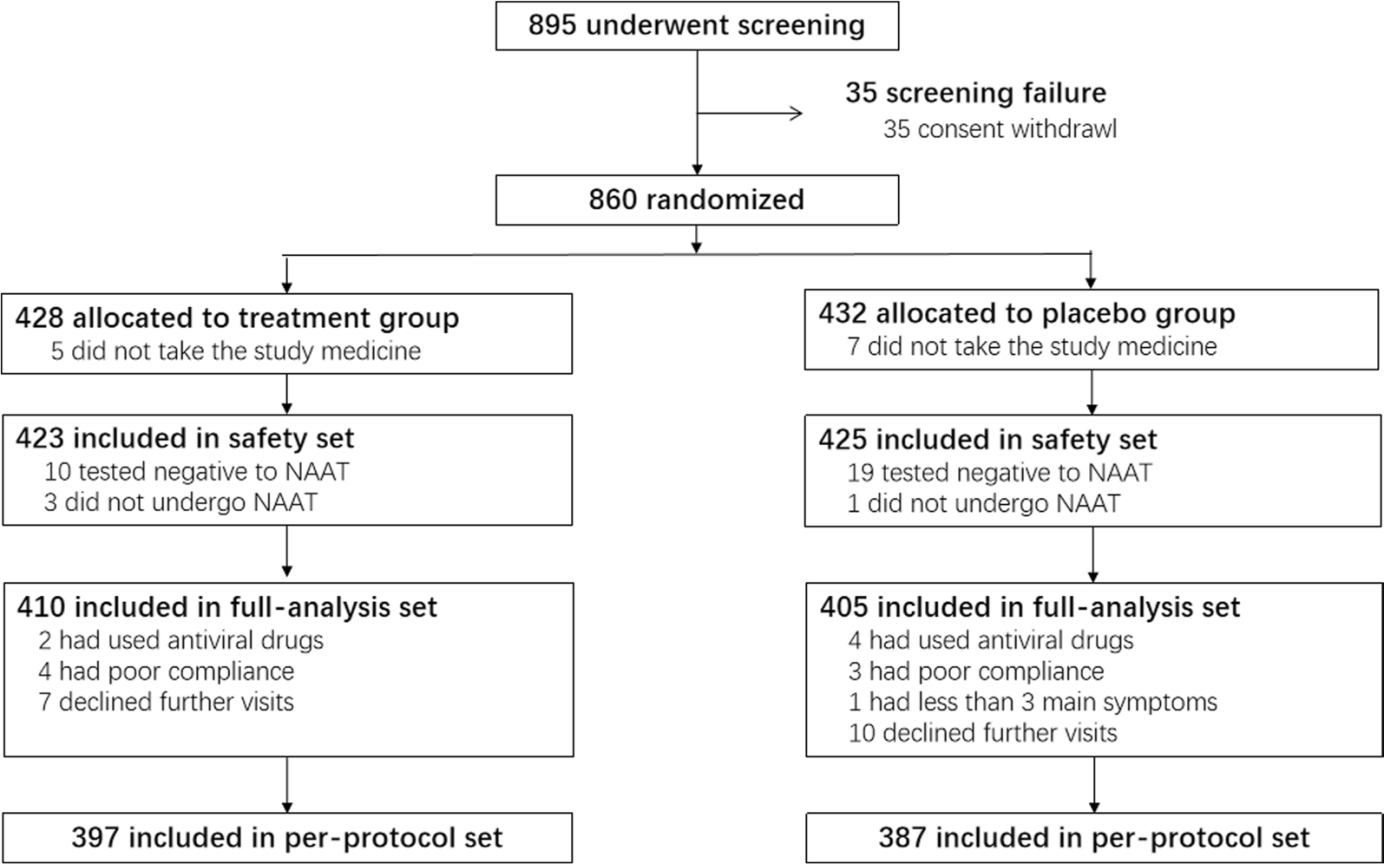
Study flow chart. FAS: full analysis set; PPS: per protocol set

The study flow chart is demonstrated in Fig. 1. Of 895 patients assessed for eligibility, 35 withdrew consent and 33 did not undergo or tested negative to NAAT (Additional file 1: Table S2). Finally, the full-analysis set consisted of 410 patients in treatment group and 405 in placebo group, and the per-protocol set of 397 patients in treatment group and 387 in placebo group. Patient’s distribution stratified by countries is shown in Additional file 1: Table S3. 93.2% and 95.1% in LHQW group and placebo group had a compliance of 80-120%, respectively.

**Table 2:** Comparison of the primary and secondary endpoints

Time to the resolution of symptoms	LHQW treatment group		Placebo group		HR (95%CI)	*P* value
	No. (%) of responders	Duration (days), Median (95% CI)	No. (%) of responders	Duration (days), Median (95% CI)		
Primary endpoint (Full-analysis set)						
Nine major symptoms *	356 (86.8%)	4.0 (3.8–4.8)	291 (71.9%)	6.7 (5.9–7.9)	1.63 (1.39–1.90)	< 0.001
Primary endpoint (Per-protocol set)						
Nine major symptoms *	349 (87.9%)	4.0 (3.8–4.7)	283 (73.1%)	6.6 (5.8–7.7)	1.64 (1.40–1.92)	< 0.001
Secondary endpoints (full-analysis set)						
Stuffy or runny nose	297 (94.9%)	2.8 (2.3–3.0)	278 (87.1%)	3.7 (2.9–4.0)	1.43 (1.21–1.69)	< 0.001
Sore throat	318 (96.4%)	2.0 (1.9–2.4)	309 (90.4%)	2.6 (2.2–3.0)	1.43 (1.22–1.67)	< 0.001
Cough	318 (90.1%)	3.2 (2.9–3.7)	259 (73.6%)	4.9 (4.5–5.9)	1.68 (1.43–1.99)	< 0.001
Shortness of breath	77 (95.1%)	2.0 (1.1–2.8)	81 (89.0%)	2.7 (1.7–2.9)	1.19 (0.87–1.62)	0.279
Low energy or tiredness	237 (96.0%)	1.3 (1.1–1.8)	232 (91.7%)	1.9 (1.7–2.2)	1.40 (1.17–1.68)	< 0.001
Myalgia	216 (97.7%)	1.5 (1.2–1.8)	203 (92.3%)	2.0 (1.6–2.2)	1.40 (1.15–1.70)	< 0.001
Headache	194 (96.5%)	1.4 (1.1–1.7)	197 (95.6%)	1.8 (1.4–1.9)	1.15 (0.94–1.40)	0.180
Chills or shivering	109 (97.3%)	1.0 (0.9–1.3)	109 (94.8%)	1.3 (1.0–1.6)	1.23 (0.95–1.61)	0.120
Feeling hot or feverish	184 (97.9%)	1.0 (1.0–1.3)	188 (94.5%)	1.3 (1.0–1.7)	1.31 (1.07–1.60)	0.010
Normalized body temperature within 2 weeks	80 (98.8%)	1.0 (0.8–1.4)	73 (98.6%)	1.6 (1.3–1.9)	1.37 (1.00–1.89)	0.052

*The nine major symptoms consisted of stuffy or runny nose, sore throat, cough, shortness of breath, low energy or tiredness, myalgia, headache, chills or shivering, feeling hot or feverish which occurred within 12 hours prior to screening

Responders denoted the patients who had a sustained improvement or resolution of the symptoms

**Table 3:** Comparison of the main treatment-emergent adverse events in the safety set

	Treatment group	Placebo group	Total
No.	423	425	848
Overall n (%)	30 (7.1)	33 (7.8)	63 (7.4)
Laboratory tests, n (%)	14 (3.3)	17 (4.0)	31 (3.7)
Psychiatric disorders, n (%)	3 (0.7)	5 (1.2)	8 (9.4)
Cutaneous or subcutaneous disorders, n (%)	4 (0.9)	1 (0.2)	5 (5.9)
Neurological diseases, n (%)	2 (0.5)	2 (0.5)	4 (4.7)
Gastrointestinal disorders, n (%)	3 (0.7)	1 (0.2)	4 (4.7)
Vascular or lymph disorders, n (%)	2 (0.5)	2 (0.5)	4 (4.7)
Cardiac disorders, n (%)	1 (0.2)	2 (0.5)	3 (3.5)
Metabolic and nutritional disorders, n (%)	2 (0.5)	0 (0)	2 (2.4)
Nasal disorders, n (%)	2 (0.5)	0 (0)	2 (2.4)
Infectious disorders, n (%)	1 (0.2)	1 (0.2)	2 (2.4)
Respiratory disorders, n (%)	0 (0)	1 (0.2)	1 (1.2)
Musculoskeletal and connective tissue disorder, n (%)	0 (0)	1 (0.2)	1 (1.2)
Immune disorders, n (%)	0 (0)	1 (0.2)	1 (1.2)
TEAE, n (%)	30 (7.1)	33 (7.8)	63 (7.4)
ADR, n (%)	3 (0.7)	4 (0.9)	7 (0.8)
SAE, n (%)	0 (0)	0 (0)	0 (0)
SADR	0 (0)	0 (0)	0 (0)
TEAE leading to study drug discontinuation	0 (0)	2 (0.5)	2 (0.2)
ADR leading to study drug discontinuation	0 (0)	0 (0)	0 (0)

*TEAE* treatment-emergent adverse event, *ADR* adverse drug reaction, *SADR* severe adverse drug reaction, *SAE* serious adverse event

Overall, patients were aged 37 years and the gender distribution was balanced. 61.1% were from mainland China, following by Thailand (28.2%). Forty-three and 21 patients were from Vietnam and the Philippines, respectively. 89.2% had received full vaccination. The most common symptom was cough (86.5%), followed by sore throat (82.5%), and stuffy or runny nose (77.5%). Only 21.1% had shortness of breath. Both groups had comparable demographic characteristics, symptoms and vaccination status (all *P*>0.05) in full-analysis set (Table 1) and per-protocol set (Additional file 1: Table S4). There was no significant difference in the proprotion of patients with Charlson comorbidity index being greater than 2.0 between the two groups. The baseline levels of vaccination status prior to enrollment were comparable between the two groups.

**Fig. 2:**
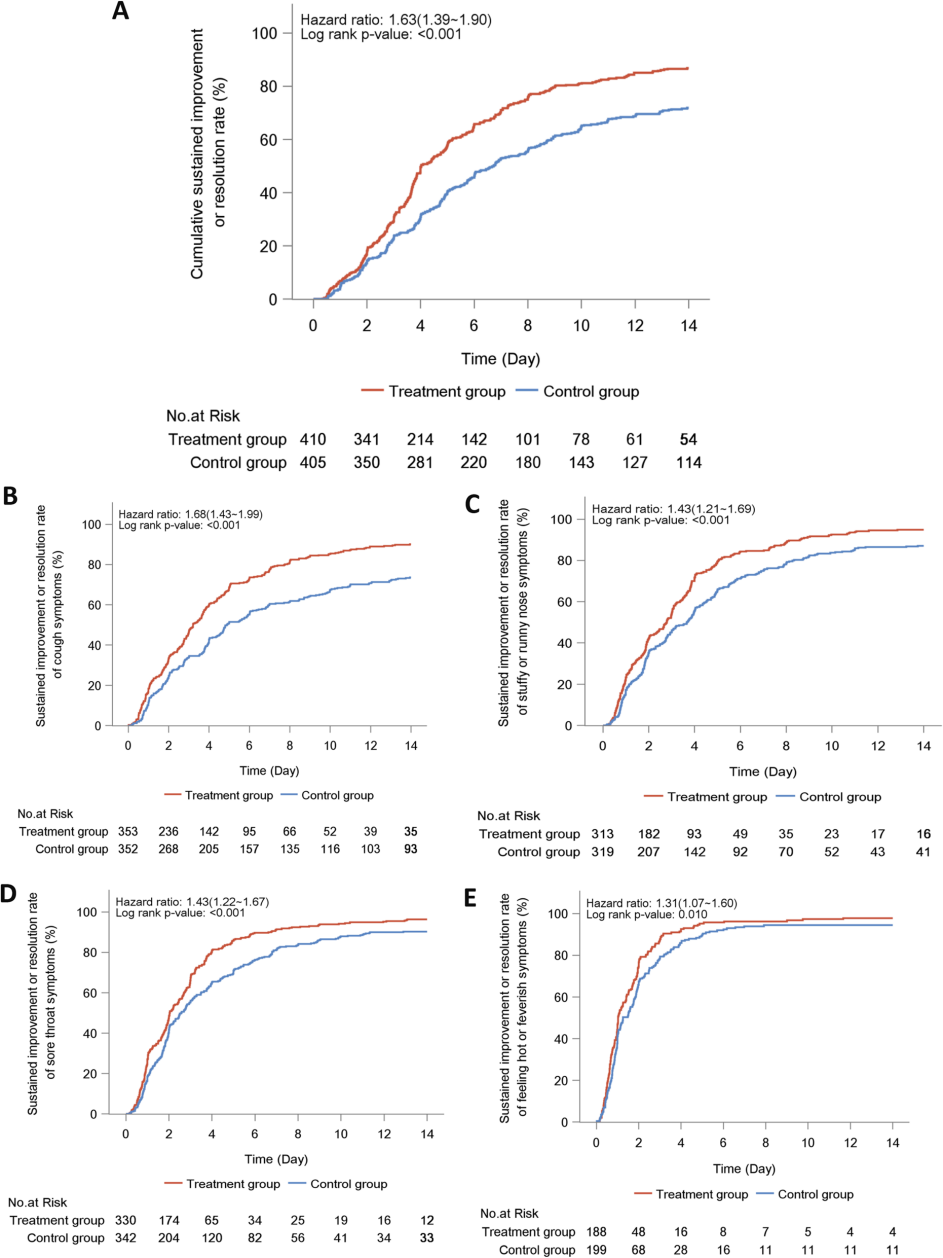
The time to the resolution of the four major symptoms in the treatment group (red curve) and placebo group (blue curve) according to the full-analysis set. **A** Time to resolution of the nine major symptoms; **B** Time to resolution of cough; **C** Time to resolution of stuffy or runny nose; **D** Time to resolution of sore throat; **E** Time to resolution of feeling hot or feverish; Shown in the figures are the median value and 95% confidence intervals (95%CI)

Treatment with LHQW significantly shortened the primary endpoint in full-analysis set (4.0 vs. 6.7 days, HR: 1.63, 95% CI 1.39–1.90) and per-protocol set (4.0 vs. 6.6 days, HR: 1.64, 95% CI 1.40–1.92) (Fig. 2). The curve of LHQW group began to diverge from that of placebo group at month 2 and thereafter. In full-analysis set, a markedly higher proportion of patients in LHQW group achieved major symptom resolution within 14 days (86.8% vs. 71.9%, *P*< 0.001). These findings remained robust when stratified by the country, sex, age interval, prior vaccination, concomitant use of other Chinese herbs, or the duration from symptom onset to randomization (Additional file 1: Table S5). Furthermore, findings of the proportion of patients who achieved major symptom resolution within 14 days in per-protocol set were not materially altered (87.9% vs. 73.1%, *P*< 0.001) (Additional file 2: Fig. S1).

**Fig. 3:**
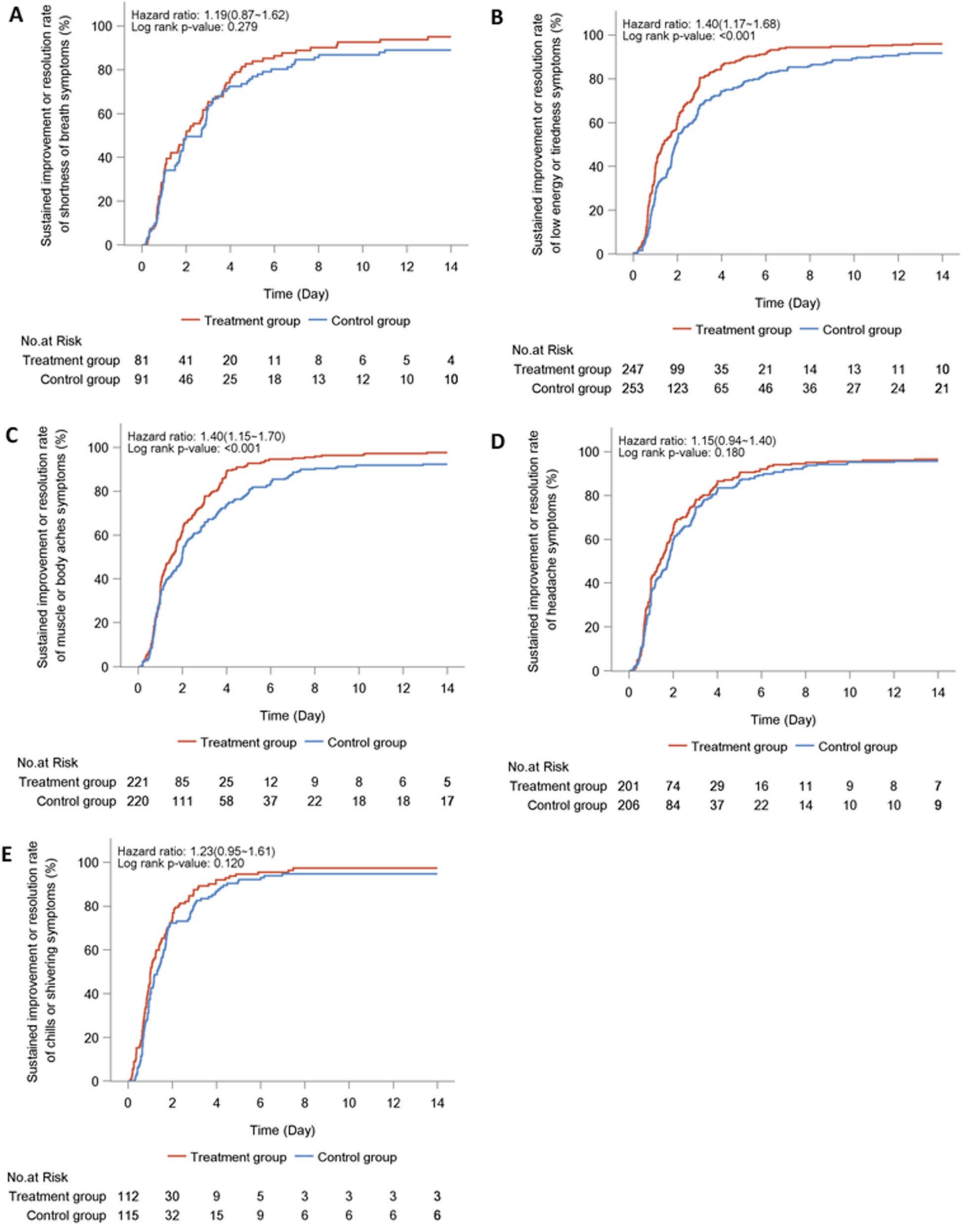
The time to the resolution of the other major symptoms in the treatment group (red curve) and placebo group (blue curve) according to the full-analysis set. **A** Time to resolution of shortness of breath; **B** Time to resolution of low energy or tiredness symptoms; **C** Time to resolution of myalgia; **D** Time to resolution of headache; **E** Time to resolution of chills or shivering

The duration to resolution of individual major symptoms is shown in Table 2. LHQW was associated with a markedly shorter median time to sustained improvement or resolution of stuffy or runny nose (2.8 vs. 3.7 days, HR: 1.43, 95% CI 1.21–1.69), sore throat (2.0 vs. 2.6 days, HR: 1.43, 95% CI 1.22–1.67), cough (3.2 vs. 4.9 days, HR: 1.68, 95% CI 1.43–1.99), and feeling hot or feverish (1.0 vs. 1.3 days, HR: 1.31, 95% CI 1.07–1.60) (Fig. 2), low energy or tiredness (1.3 vs. 1.9 days, HR: 1.40, 95% CI 1.17–1.68), and myalgia (1.5 vs. 2.0 days, HR: 1.40, 95% CI 1.15–1.70). However, the difference in time for shortness of breath (2.0 vs. 2.7 days, HR: 1.19, 95% CI 0.87–1.62), headache (1.4 vs. 1.8 days, HR: 1.15, 95% CI 0.94–1.40), and chills or shivering (1.0 vs. 1.3 days, HR: 1.23, 95% CI 0.95–1.61) did not differ significantly (Fig. 3). Similar findings applied to the per-protocol analysis.

Furthermore, the time to sustained improvement or resolution of anosmia and ageusia (2.7 vs. 3.6 days, HR: 1.21, 95% CI 0.89–1.64) or gastrointestinal symptoms (1.7 vs. 1.8 days, HR: 1.17, 95% CI 0.89–1.54) did not differ considerably, albeit a nominally shorter time to the return of normal body temperature (1.0 vs. 1.6 days, HR: 1.37, 95% CI 1.00–1.89) in LHQW group (Additional file 1: Table S6; Additional file 3: Fig. S2). However, significantly more patients in LHQW group had sustained improvement or resolution of all symptoms combined (85.1% vs. 71.1%, *P*<0.001).

**Fig. 4:**
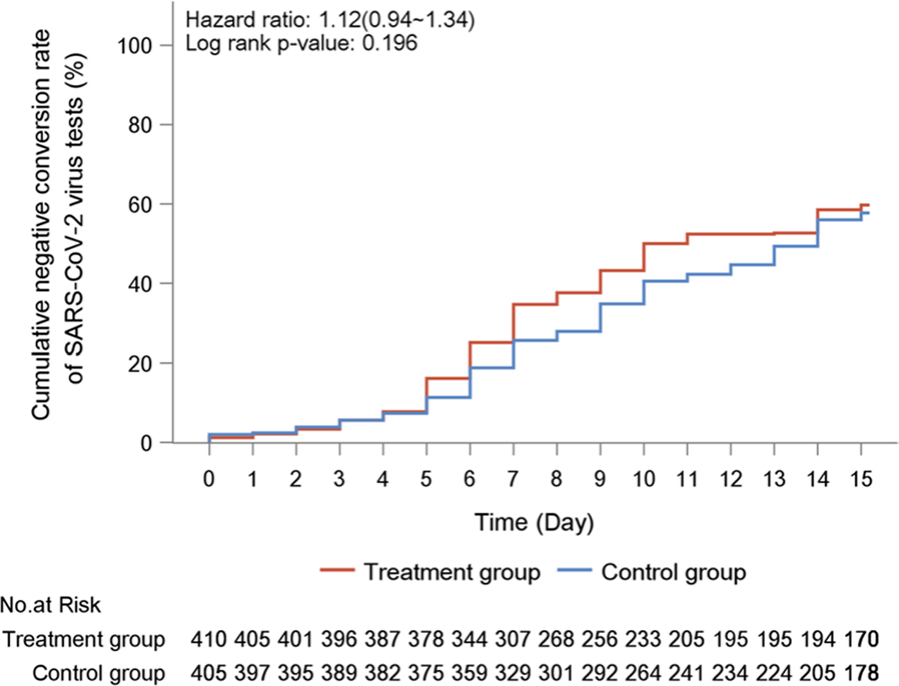
Cumulative negative conversion rate of SARS-CoV-2 NAAT findings in the treatment group (red curve) and placebo group (blue curve) according to the full-analysis set. No significant difference was shown between the two curves for each individual time points

Treatment with LHQW did not accelerate the negative conversion of NAAT findings (10.5 vs. 14.0 days, HR: 1.12, 95% CI 0.94–1.34) (Fig. 4). Only 15 patients in LHQW group and 9 in placebo group had radiologic evidence of pneumonia at baseline, and the rate of chest imaging improvement was nominally higher in LHQW group (66.7% vs. 33.3%, *P*=0.206). No patient progressed into severe or critical illness, or died within 14 days.

The safety set consisted of 423 patients in LHQW group and 425 in placebo group. Overall, treatment-associated AE was reported in 7.4% of patients, with no significant between-group difference (7.1% vs. 7.8%, *P*=0.709) (Table 3, Additional file 1: Table S7). Overall, the most common events consisted of psychiatric disorders (9.4%), cutaneous or subcutaneous disorders (5.9%). Adverse drug responses were reported in 0.7% and 0.96% of patients, respectively. Two patients in placebo group discontinued treatment due to AEs whereas no patient in LHQW group discontinued treatment. No serious AE was reported.

**Fig. 5:**
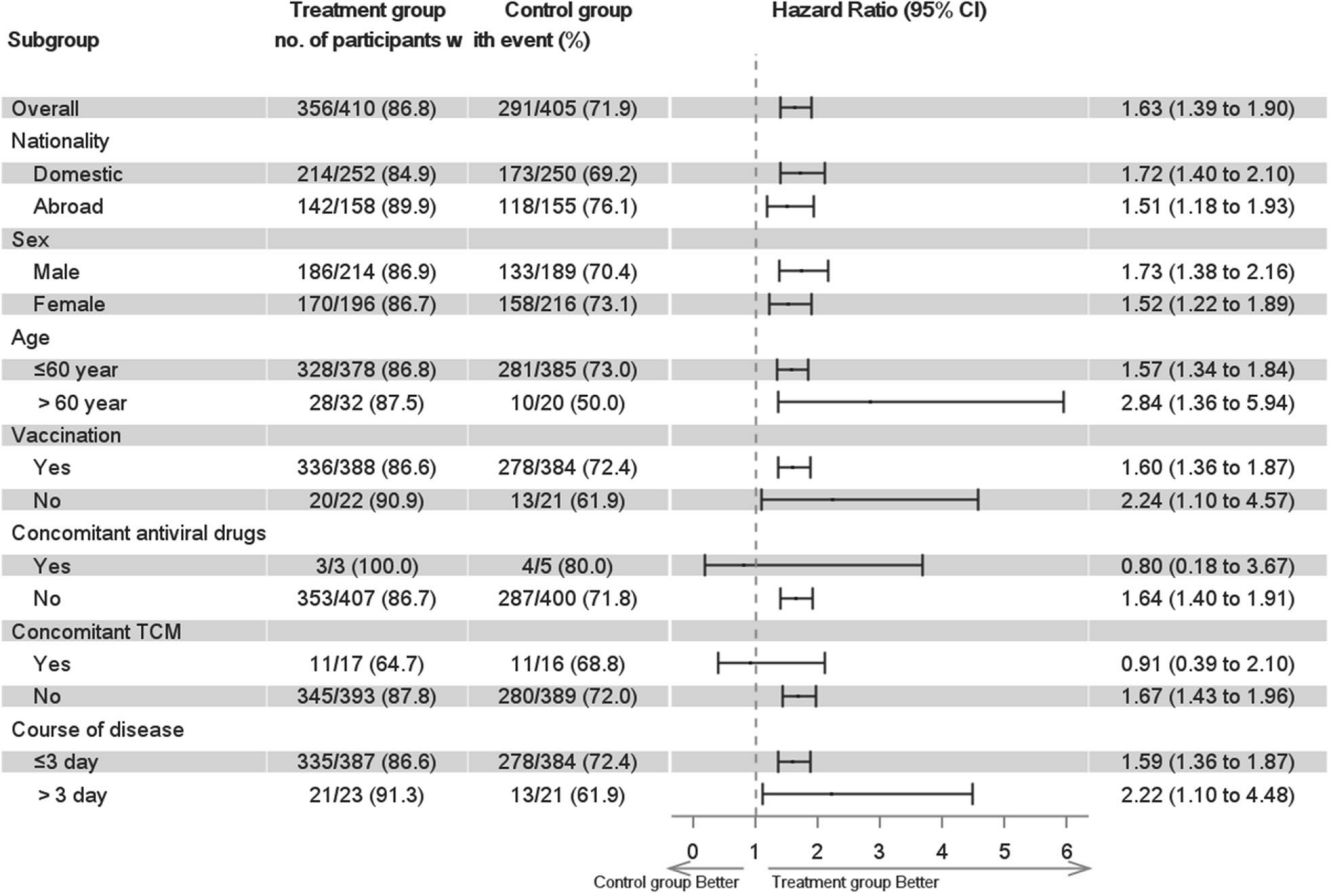
Subgroup analysis of the therapeutic effect of the primary endpoint (the median time to sustained clinical improvement or resolution of the nine above-mentioned major symptoms as of day 14)

Subgroup analysis did not reveal any stratum that demonstrated a significant therapeutic effect of the primary endpoint favoring LHQW group (all *P*>0.05) (Fig. 5).

## Discussion

The FLOSAN study is the first international multicenter randomized controlled trial that evaluates the safety and efficacy of LHQW capsules among patients with mild-to-moderate COVID-19 in the western Pacific region. Treatment with LHQW for 14 days significantly shortened the time to sustained improvement or resolution of most cardinal symptoms and all symptoms combined. LHQW did not markedly accelerate the negative conversion of NAAT findings. No patient progressed into severe or critical illness or died. LHQW was well tolerated (Additional files 2 and 3.

With greater scientific rigor in the study design, findings of the FLOSAN study echoed the principal results of our previously published multicenter randomized study [15]. The difference in the median time to symptom resolution in the FLOSAN study might be primarily due to the different viral strain (Omicron variant vs. Ancestral strain) and vaccination status (Partly/fully vaccinated vs. Unvaccinated). Although the between-group difference in the primary efficacy endpoint did not reach our pre-defined level (3.0 days), the therapeutic effects were mostly consistent for most major symptoms that could influence the quality-of-life. This was more prominent for respiratory symptoms, which were elicited by the Omicron variant that conferred a high binding affinity to the upper airway epithelium [18].

Our findings were consistent with other published studies pertinent to Chinese herbs for treatment of COVID-19. Several meta-analyses, each with different sample sizes and studies with different design, have concluded the therapeutic benefits of LHQW capsules for mild-to-moderate COVID-19 [19]. These mainly included the increased rate of clinical recovery, decreased probability of progression into severe or critical illness and the similar safety profile compared with control group. The effect of LHQW in preventing disease progression cannot be evaluated in our study, possibly because we have recruited the study participants infected with the Omicron variant who were overall younger and did not have multiple comorbidities compared with those enrolled in our previous multicenter trial [20–22]. Although another previous study demonstrated the prophylactic effects of LHQW among close contacts of infected individuals [23], and the *in vitro* study has demonstrated anti-viral effects [13], the present study did not suggest any therapeutic effect of LHQW capsules in accelerating viral clearance.

LHQW capsules were safe among patients with COVID-19, evidenced by the similar safety profile as compared with placebo group, which echoed with our [15] and others findings [23]. The spectrum of AE and treatment-emergent AE did not reveal additional major safety signals associated with LHQW. No patient reported SAE, reaffirming the safety for clinical application.

The main strengths included the international multicenter trial which has recruited a large sample size of patients from four countries of the western Pacific region, with no major differences in the therapeutic responses and safety profiles across the countries. Therefore, the findings were likely generalizable to patients from Asia. The double-blind study design with matching placebo has provided more solid evidence pertaining to the efficacy of LHQW capsules. Few clinical trials of the existing marketed drugs have been conducted during the Omicron outbreak, when the current trial was initiated.

Our findings have further validated the efficacy of LHQW capsules for Omicron, the prevailing variant of SARS-CoV-2 circulating globally. This is important because of the scarcity of marketed medications and the large number of vulnerable population within mainland China, where some of the repurposed herbs with notable therapeutic potentials have already been marketed. LHQW capsules might help reduce healthcare burden during the surge of Omicron wave among community-dwelling patients with mild-to-moderate COVID-19.

Some limitations should also be considered. First, there existed an imbalance in the sample size within individual countries, although we have not identified the signals indicating differential therapeutic responses across the nations. However, our findings were not materially altered in the main study outcomes when stratified by the countries (China vs. The rest combined). Second, the relatively short duration of observation cannot provide further evidence of the longer-term efficacy of LHQW (e.g. at day 28 or beyond). Third, caution should be exercised when extrapolating our conclusions to other populations, such as those with multiple comorbidities or immunosuppression. Furthermore, our study cannot directly address the molecular mechanisms underlying the efficacy of LHQW for the treatment of COVID-19. Finally,we did not employ the stratified randomization scheme. However, randomization was well balanced across the different metrics that could have affected the study outcomes (e.g. sex, age, duration of symptoms, disease severity). In light of the difference in the study regions and study sites, we have adopted the central block randomization scheme, and the results demonstrated balanced distribution of the demographic and other baseline disease severity metrics.

## Conclusion

LHQW capsules are effective and safe for mild-to-moderate COVID-19 via accelerating symptom resolution and clinical recovery for mild-to-moderate COVID-19. LHQW might be worthwhile for patients who could now be managed within the community.

### Supplementary Information

**Additional file 1:** Online supplementary tables and figures.

**Additional file 2: Figure S1.** The percentage of patients who achieved sustained improvement or resolution of the main symptoms in the per-protocol set. Shown in the figures are the bars of the treatment group (red) and placebo group (blue).

**Additional file 3: Figure S2**. The time to the resolution of additional symptoms evaluated in the treatment group (red curve) and placebo group (blue curve) according to the full-analysis set. A Time to resolution of anosmia and ageusia; B Time to the return of body temperature to normal levels; C Time to resolution of gastrointestinal symptoms.

## Data Availability

Data could be shared after publication after consultation with the corresponding authors.

## Abbreviations

COVID-19: Coronavirus disease 2019
SARS-CoV-2: Severe acute respiratory syndrome coronavirus 2
LHQW: Lianhuaqingwen
FAS: Full-analysis set
PPS: Per protocol set
SS: Safety set
95%CI: 95% confidence interval
HR: Hazards ratio
