# Supplementary material for: Effects of Lianhuaqingwen Capsules in adults with mild-to-moderate coronavirus disease 2019: an international, multicenter, double-blind, randomized controlled trial
Source: Virol J. 2023 Nov 28;20:277. doi: 10.1186/s12985-023-02144-6 (PMC10685492; doi:10.1186/s12985-023-02144-6)
Supplement: Supplementary file 1 — Additional file 1: Online supplementary tables and figures. [file 12985_2023_2144_MOESM1_ESM.docx]

**Online supplement**

**Effects of Lianhuaqingwen Capsules in adults with mild-to-moderate coronavirus disease 2019: An international, multicenter, double-blind, randomized controlled trial**

**Methods**

***Inclusion criteria***

(1) Adult patients with mild-to-moderate COVID-19 (according to the WHO criterion), who tested positive to SARS-CoV-2 rapid antigen test (RAT) or RT-PCR nucleic acid amplification test (NAAT) for confirmation of SARS-CoV-2 infection;

(2) Adult males and non-pregnant females, aged between 18 and 70 years (including 18 and 70 years);

(3) The time interval between the onset of symptoms and screening not exceeding 4 days.

The occurrence of signs and symptoms of COVID-19 might appear on the 2^nd^ to 14^th^ days after the exposure to SARS-CoV-2. The onset of symptoms was defined having at least one of the following symptoms: cough, stuffy or runny nose, sore throat, shortness of breath (difficulty in breathing), low energy or tiredness, muscle or body aches, headache, chills or shivering, feeling hot or feverish, nausea, vomiting, diarrhea, anosmia (new-onset loss of smell), and ageusia (new-onset loss of taste).

(4) Having at least 3 of the 9 major symptoms (stuffy or runny nose, sore throat, cough, shortness of breath (difficulty breathing), low energy or tiredness, muscle or body aches, headache, chills or shivering, feeling hot or feverish) which occurred within 12 hours before screening.

(5) Could understand and comply with the requirements of the protocol, and could provide a signed written informed consent.

***Exclusion criteria***

(1) Patients who met the COVID-19 diagnostic criteria of asymptomatic disease, severe diseases or critical illness.

(2) Based on the assessment of investigator, patients who had any of the following serious chronic systemic diseases that might affect the efficacy evaluation and disease regression:

① Poorly controlled diabetes (randomized blood glucose >11.1 mmol/L within 7 days prior to enrolment or glycated hemoglobin ≥8% within the last 3 months in the case of receiving maintenance standardized glucose-lowering therapy);

② Poorly controlled hypertension (systolic blood pressure ≥ 140 mmHg or diastolic blood pressure ≥ 90 mmHg despite regulated use of antihypertensive medication);

③ Chronic lung diseases, including chronic obstructive pulmonary disease (COPD), asthma, interstitial lung disease, cystic fibrosis and pulmonary hypertension;

④ Malignancy, severe cardiovascular diseases (heart failure, cardiomyopathy, dementia, mental disorders, etc.), diseases that seriously affect the immune system, etc.

⑤ Chronic kidney disease (including chronic kidney disease stages 3-5), chronic liver disease (e.g. liver fibrosis, cirrhosis, or serum AST and/or ALT ≥ 3 times the upper limit of normal).

⑥ Severe obesity: BMI ≥ 35 kg/m^2^.

(3) Patients with other known infections.

(4) Patients with a history of alcohol or drug abuse (other than cannabis use) within 1 year prior to enrollment.

(5) Those participated in other clinical trials within 1 month prior to screening. If the investigational product (IP) has a long half-life, the time interval should be more than 5 half-lives from the last administration to screening of the study.

(6) Individuals with a known or suspected history of allergy or hypersensitivity to the IP and its excipients.

(7) Female patients who were pregnant, breastfeeding or within 2 weeks of delivery.

(8) Patients who, in the opinion of the investigator, would not be suitable to participate in the study due to a possible breach of the trial protocol or any other circumstances affecting their safety assessment.

***Packaging and labeling of the investigational products***

The IPs were assigned the corresponding drug numbers and the labels. The outer packaging of IP and placebo should be consistent, and the outer packaging should be labeled with text of drug number, the name of drug, the indication of use, storage conditions, batch number, shelf-life, expiration date, manufacturer and "for clinical research use only".

***Prohibited concomitant medications***

(1) Hydroxychloroquine and lopinavir/ritonavir, remdesivir, systemic glucocorticoids and antibodies against cytokines already approved for COVID-19 are prohibited by the protocol during the study (from enrollment/pre-dose to the end-of-study)

(2) Chinese herbal preparations or proprietary Chinese medicines (herbs and botanical drugs) that contain the same ingredients or have similar efficacy to LHQW are prohibited by the protocol.

**Allowed concomitant medications**

(1) Symptomatic treatment such as antipyretic analgesia, adequate nutrition and appropriate rehydration in patients with mild COVID-19 were allowed according to the study protocol. Antipyretic and analgesic acetaminophen could be given as symptomatic treatment when the body temperature exceeded 38.5°C.

(2) Conventional drugs used for the treatment of COVID-19 during the study period must be recorded on the case report form.

(3) Any combination therapy during the study period must be recorded on the case report form.

**Primary Efficacy Endpoint**

The primary endpoint was the median time to sustained improvement or resolution of 9 major symptoms. (during the 14-day observation period)

The time to sustained improvement or resolution of symptoms was defined as the primary symptoms, including respiratory symptoms (stuffy or runny nose, sore throat, cough, shortness of breath (difficulty breathing)) and systemic symptoms (low energy or tiredness, myalgia, headache, chills or shivering, feeling hot or feverish) are less than or equal to mild (score is 1 or 0) and are stable for at least 24 hours;

Temperature and symptom evaluation was recorded in the diary cards, with patients evaluating the severity of their symptoms every morning and evening.

**Secondary Efficacy Endpoints**

(1) The proportion of patients with sustained improvement or resolution of 9 major symptoms; (within the 14-day observation period)

(2) The median time to sustained improvement or resolution of individual symptoms of the 9 primary symptoms; (within the 14-day observation period)

(3) The median time to the onset of antipyretic effect and return to normal body temperature; (within the 14-day observation period)

- Definition of return to normal body temperature: axillary temperature ≤ 37.0°C or oral temperature ≤ 37.3°C for at least 24 hours.

(4) The median time to sustained improvement or resolution of gastrointestinal symptoms; (within the 14-day observation period)

(5) The median time to sustained improvement or resolution of anosmia and ageusia; (within the 14-day observation period)

(6) The proportion of patients with marked improvement or resolution of all clinical symptoms; (within the 14-day observation period)

(7) The time to and the rate of negative conversion of NAAT for SARS-CoV-2. (within the 14-day observation period, planned testing dates: days 0, 7, 10, 14)

(8) The rate of improvement in chest imaging (CT/chest x-ray) findings, with the analysis being conducted among patients who had radiologic evidence of pneumonia at baseline (within the 14-day observation period) (evaluation at conditioned centers)

(9) Incidence of severe/critical COVID-19. (within the 14-day observation period)

- Severe COVID-19: clinical signs indicative of pneumonia (fever, cough, difficulty breathing, shortness of breath), plus one of the following signs: respiratory rate >30 breaths per minute, severe dyspnea, or SpO_2_<90% at room air.
- Critical COVID-19: acute respiratory distress syndrome requiring mechanical ventilation, sepsis, infectious shock, combined with other organ failure requiring ICU care and other critical conditions.

(10) Incidence of COVID-19-related and all-cause mortality. (within the 14-day observation period)

***Safety Endpoints***

Safety endpoints should be evaluated from first dosing to end of follow-up, including the follows

(1) Vital signs (body temperature, heart rate, blood pressure, respiration), physical examination.

(2) Changes in laboratory test: blood routine (red blood cell count, hemoglobin, platelets, etc.), routine urine, liver and kidney function (glutamate transaminase, glutamic oxalacetic transaminase, total bilirubin, creatinine, urea nitrogen), cardiac enzymes (lactate dehydrogenase, creatine kinase, creatine kinase isoenzyme);

(3) Twelve-lead electrocardiogram;

(4) Adverse event rate and serious adverse event rate.

If the clinical conditions worsened, regular examination should be performed until the test items became normal or returned to the baseline levels or the investigator considered that follow-up would not be necessary.

***Blinding and unblinding***

(1) Blinding design: Double-blind, that is, both the researchers and patients were in a blinded state during the whole clinical trial.

(2) Blinding: the on-site blinding should be attended by the statistical personnel and the personnel irrelevant to this clinical trial. The drug number should be pasted on the label, and the blinding records of this process should be documented.

(3) Breaking the Study Blind: Only in the event of a serious adverse event, when the investigator deemed that the subject cannot be adequately treated without knowing the identity of the study medication, could the medication code be broken for a particular subject. Every effort must be made to contact the medical monitor prior to breaking the code. If this would not be possible and the situation was emergent, the investigator could break the code (from the Emergency Unblinding Envelope) and contact the medical monitor as soon as possible thereafter. The event should be well documented.

(4) Regulations on unblinding: The unblinding should be performed after the statistical analysis plan and data review report was finalized and the database was locked. The application should be made by the project statistician, and then the treatment group corresponding to the random number should be announced after the approval of the sponsor, so as to conduct the statistical analysis after grouping all data.

***Analysis of the primary efficacy endpoint***

The time to sustained alleviation for 9 main symptoms within 2 weeks was be analyzed by Log-rank test. The percentage of patients being censored, the quartile and 95% confidence interval were reported respectively, and visualized with Kaplan-Meier curve. Analysis was based on the FAS and PPS.

***Analysis of the secondary efficacy endpoints***

(1) The proportion of patients with alleviation of 9 main symptoms within 2 weeks was analyzed by χ^2^ test or Fisher’s exact probability method to compare the differences between the two groups.

(2) The time to sustained alleviation of single symptom (9 main symptoms) within 2 weeks was analyzed by using the Kaplan-Meier method. The percentage of patients being censored, the quartile and 95% confidence interval were reported respectively, and visualized with Kaplan-Meier curve.Log-rank test will be used for comparison between the two groups.

(3) The time to the alleviation of fever within 2 weeks was analyzed by using the Kaplan-Meier method. The percentage of patients being censored, the quartile and 95% confidence interval were reported respectively, and visualized with Kaplan-Meier curve. Log-rank test will be used for comparison between the two groups.

(4) The time to the alleviation of digestive symptoms within 2 weeks was analyzed by using the Kaplan-Meier method. The percentage of patients being censored, the quartile and 95% confidence interval were reported respectively, and visualized with Kaplan-Meier curve. Log-rank test will be used for comparison between the two groups.

(5) The time to the alleviation of decreased sense of smell and taste symptoms within 2 weeks was analyzed by using the Kaplan-Meier method. The percentage of patients being censored, the quartile and 95% confidence interval were reported respectively, and visualized with Kaplan-Meier curve. Log-rank test will be used for comparison between the two groups.

(6) The proportion of patients with alleviation of all clinical symptoms within 2 weeks was analyzed by χ^2^ test or Fisher’s exact probability method to compare the differences between the two groups.

(7) The duration of viral shedding within 2 weeks was analyzed by using the Kaplan-Meier method. The percentage of patients being censored, the quartile and 95% confidence interval were reported respectively, and visualized with Kaplan-Meier curve. Log-rank test will be used for comparison between the two groups.

(8) The rate of reduction in viral shedding within 2 weeks was analyzed by χ^2^ test or Fisher’s exact probability method to compare the differences between the two groups.

(9) The improvement rate of imaging (CT / chest X-ray) within 2 weeks was analyzed by χ^2^ test or Fisher’s exact probability method to compare the differences between the two groups.

(10) The incidence of severe and critical Covid-19 within 2 weeks was analyzed by χ 2 test or Fisher’s exact probability method to compare the differences between the two groups.

(11) The incidence of death and all-cause death related to Covid-19 within 2 weeks was analyzed by χ^2^ test or Fisher’s exact probability method to compare the differences between the two groups.

All of the above-mentioned analyses were based on the full-analysis set.

***Analysis of Safety***

(1) All AEs recorded on the eCRF were coded by using the Medical Dictionary for Regulatory Activities (MedDRA) dictionary.

(2) All AE summaries were restricted to treatment-emergent adverse events (TEAEs), which are defined as the AEs that occurred after dosing and the existing AEs that worsened during the study. Summaries of patient’s incidence of TEAEs, drug-related TEAEs, SAE and TEAEs leading to discontinuation of the study were also presented.

(3) Patient’s incidence and the total number of unique TEAEs, drug-related TEAEs, SAE and TEAEs leading to discontinuation of the study by system organ class (SOC), preferred term (PT) and severity were demonstrated.

(4) The list would include TEAEs, drug-related TEAEs, SAE and TEAEs leading to discontinuation of the study.

(5) The shifting of status was demonstrated by using a table showing the conditions from baseline to the post-baseline value according to the abnormality assessed by investigator, and were presented as the laboratory test, electrocardiograms and physical examination.

(6) All laboratory data, electrocardiograms and physical examination were listed according to the patients and visits.

(7) Vital signs data and changes from baseline in vital signs were summarized by stratifying the visits using standard descriptive statistics.

**RESULTS**

**Table S1. A list of the participating center and the number of patients enrolled**

| **Site No.** | **Country** | **Hospital** | **Principal investigator** | **No. enrolled** |
| --- | --- | --- | --- | --- |
| 2 | China | Shanghai Public Health Clinical Center | Yun Ling | 154 |
| 3 | China | The Third People's Hospital of Shenzhen | Hong-zhou Lu/ Jing Yuan | 90 |
| 4 | China | Guangzhou Eighth People’s Hospital, Guangzhou Medical University | feng-yu Hu/ Xing-hua Tan | 48 |
| 5 | China | Yunnan Provincial Infectious Disease Hospital | Xing-qi Dong | 0 |
| 6 | China | Public Health Clinical Center of Chengdu | Liang-shuang Jiang/ Yi Mao | 128 |
| 7 | China | Kunming Third People’s Hospital | Jian-jie Jiang | 8 |
| 8 | China | Tangshan Hospital of Traditional Chinese Medicine | Hui-min Sun | 10 |
| 10 | China | Hefei Binhu Hospital | Zhen Ding | 6 |
| 11 | China | The Affiliated Hospital of Changchun University of TCM | Li-xiu Zhang | 79 |
| 13 | China | First People’s Hospital of Zhengzhou City | Ren-jie Liu | 8 |
| 14 | China | Xi'an Chest Hospital | Xing Gu | 12 |
| 15 | China | Fuzhou Pulmonary Hospital of Fujian | Xiao-hong Chen | 1 |
| 101 | Thailand | Srinagarind Hospital | Piroon Mootsikapun | 112 |
| 102 | Thailand | Siriraj Hospital | Methee Chayakulkeeree | 80 |
| 103 | Thailand | Burapha University Hospital | Pureepat Arttawejkul | 60 |
| 202 | The Philippines | Dr. Jose N. Rodriguez Memorial Hospital and Sanitarium | Roxan A. Perez | 21 |
| 301 | Vietnam | Traditional Medicine Institute of Ho Chi Minh City | Truong Thi Ngoc Lan | 43 |

**Table S2. Reasons for patients not being included in the per-protocol set**

|  | **LHQW group** | **Placebo group** | **Total** |
| --- | --- | --- | --- |
| **Randomized,(N)** | 428 | 432 | 860 |
| **Did not take the study medication, n(%)** | 5(1.2) | 7(1.6) | 12(1.4) |
| **Negative NAAT findings, n(%)** | 10(2.3) | 19(4.4) | 29(3.4) |
| **Did not undergo NAAT, n(%)** | 3(0.7) | 1(0.2) | 4(0.5) |
| **Having had antiviral drugs, n(%)** | 2(0.5) | 4(0.9) | 6(0.7) |
| **Poor compliance,n(%)** | 4(0.9) | 3(0.7) | 7(0.8) |
| **Fewer than 3 main symptoms, n(%)** | 0(0) | 1(0.2) | 1(0.1) |
| **Reason for dropout** |  |  |  |
| Adverse events, n(%) | 0(0) | 1(0.2) | 1(0.1) |
| Violation of trial protocol (including poor compliance), n(%) | 0(0) | 1(0.2) | 1(0.1) |
| Subject refused further treatment, n(%) | 6(1.4) | 3(0.7) | 9(1.0) |
| Others, n(%) | 1(0.2) | 5(1.2) | 6(0.7) |

NAAT: nucleic acid amplification test

Shown are the counts (percentages).

**Table S3. A summary of analyzed population of all randomized patients across the countries**

|  | **LHQW group** | **Placebo group** | **Total** |
| --- | --- | --- | --- |
| Total | | | |
| Randomized | 428 | 432 | 860 |
| Completed | 416 | 412 | 828 |
| FAS | 410 | 405 | 815 |
| PPS | 397 | 387 | 784 |
| SS | 423 | 425 | 848 |
| Thailand | | | |
| Randomized | 126 | 126 | 252 |
| Completed | 125 | 120 | 245 |
| FAS | 125 | 122 | 247 |
| PPS | 125 | 119 | 244 |
| SS | 125 | 124 | 249 |
| Vietnam | | | |
| Randomized | 22 | 21 | 43 |
| Completed | 22 | 21 | 43 |
| FAS | 22 | 21 | 43 |
| PPS | 22 | 21 | 43 |
| SS | 22 | 21 | 43 |
| Philippine | | | |
| Randomized | 10 | 11 | 21 |
| Completed | 10 | 10 | 20 |
| FAS | 10 | 11 | 21 |
| PPS | 9 | 8 | 17 |
| SS | 10 | 11 | 21 |
| China | | | |
| Randomized | 270 | 274 | 544 |
| Completed | 259 | 261 | 520 |
| FAS | 253 | 251 | 504 |
| PPS | 241 | 239 | 480 |
| SS | 266 | 269 | 535 |

FAS: full-analysis set, PPS: per-protocol set, SS: safety set

**Table S4. Baseline demographic and clinical characteristics of the per-protocol set**

|  | **LHQW group**  **(N=397)** | **Placebo group**  **(N=387)** | **Total**  **(N=784)** |
| --- | --- | --- | --- |
| **Age (yrs), Mean±SD** | 37.9±14.62 | 36.2±13.20 | 37.0±13.95 |
| **Females, n(%)** | 193(48.6) | 210(54.3) | 403(51.4) |
| **Height (cm), Mean±SD** | 166.1±9.0 | 165.6±8.4 | 165.8±8.7 |
| **BMI (kg/m^2^), Mean±SD** | 23.6±3.7 | 23.2±3.8 | 23.4±3.8 |
| **Nationality (region)** |  |  |  |
| China, n(%) | 237(59.7) | 237(61.2) | 474(60.5) |
| Hong Kong (China), n(%) | 3(0.8) | 1(0.3) | 4(0.5) |
| Vietnam, n(%) | 22(5.5) | 21(5.4) | 43(5.5) |
| Thailand, n(%) | 117(29.5) | 110(28.4) | 227(29.0) |
| Philippine, n(%) | 9(2.3) | 8(2.1) | 17(2.2) |
| Others, n(%) | 9(2.3) | 10(2.6) | 19(2.4) |
| **Source** |  |  |  |
| Local, n(%) | 358(90.2) | 342(88.4) | 700(89.3) |
| Immigrants, n(%) | 39(9.8) | 45(11.6) | 84(10.7) |
| **Ethnicity** |  |  |  |
| Yellow, n(%) | 388(97.7) | 378(97.7) | 766(97.7) |
| Brown, n(%) | 9(2.3) | 9(2.3) | 18(2.3) |
| Others, n(%) | 0 (0) | 0 (0) | 0 (0) |
| **COVID-19 vaccination** |  |  |  |
| Primary-series, n(%) | 359(90.4) | 340(87.9) | 699(89.2) |
| Incomplete, n(%) | 16(4.0) | 26(6.7) | 42(5.4) |
| Not vaccinated, n(%) | 22(5.5) | 21(5.4) | 43(5.5) |
| **Symptoms** |  |  |  |
| Cough, n(%) | 353(86.1) | 352(86.9) | 705(86.5) |
| Sore throat, n(%) | 330(80.5) | 342(84.4) | 672(82.5) |
| Stuffy or runny nose, n(%) | 313(76.3) | 319(78.8) | 632(77.5) |
| Low energy or tiredness, n(%) | 247(60.2) | 253(62.5) | 500(61.3) |
| Myalgia, n(%) | 215(54.2) | 212(54.8) | 427(54.5) |

**Table S5. Comparison of the primary endpoint in the full-analysis set when stratified by baseline variables**

|  |  | **LHQW group** | |  | **Placebo group** | |  |  |
| --- | --- | --- | --- | --- | --- | --- | --- | --- |
| **Strata** | **Time to the resolution of events** | **No. (%) of responders** | **Duration (days), Median (95%CI)** |  | **No. (%) of responders** | **Duration (days), Median (95%CI)** | **HR (95%CI) *** | **P value** |
| Country | China | 214(84.9%) | 3.7(3.4~3.9) |  | 173(69.2%) | 6.1(5.3~7.6) | 1.72(1.40~2.10) | <0.001 |
|  | Other countries | 142(89.9%) | 5.4(5.0~6.0) |  | 118(76.1%) | 7.4(5.9~8.5) | 1.51(1.18~1.93) | <0.001 |
| Sex | Males | 186(86.9%) | 3.9(3.7~4.4) |  | 133(70.4%) | 6.8(5.5~8.1) | 1.73(1.38~2.16) | <0.001 |
|  | Females | 170(86.7%) | 4.8(3.9~5.7) |  | 158(73.1%) | 6.5(5.6~8.0) | 1.52(1.22~1.89) | <0.001 |
| Age | ≤60 years | 328(86.8%) | 4.2(3.8~4.9) |  | 281(73.0%) | 6.6(5.8~7.9) | 4.20 (3.80~4.90) | <0.001 |
|  | >60 years | 28(87.5%) | 3.7(2.8~4.4) |  | 10(50.0%) | NA(3.8~NA) | 2.84(1.36~5.94) | 0.004 |
| Receipt of COVID-19 vaccines | Vaccinated | 336(86.6%) | 4.3(3.9~4.9) |  | 278(72.4%) | 6.7(5.9~7.9) | 1.60(1.36~1.87) | <0.001 |
|  | Unvaccinated | 20(90.9%) | 3.6(2.7~4.0) |  | 13(61.9%) | 4.8(2.8~NA) | 2.24(1.10~4.57) | 0.023 |
| Concomitant use of other Chinese herbs | Yes | 11(64.7%) | 6.6(3.6~NA) |  | 11(68.8%) | 6.7(3.8~NA) | 0.91(0.39~2.10) | 0.827 |
|  | No | 345(87.8%) | 4.0(3.8~4.6) |  | 280(72.0%) | 6.7(5.8~7.9) | 1.67(1.43~1.96) | <0.001 |

Other countries included Vietnam, Thailand, and the Philippines.

Responders denoted the patients who had a sustained improvement or resolution of the symptoms.

NA: not applicable

**Table S6. Comparison of the other clinically relevant endpoints in the full-analysis set**

|  | **LHQW group** | |  | **Placebo group** | |  |  |
| --- | --- | --- | --- | --- | --- | --- | --- |
| **Time to the resolution of events** | **No. (%) of responders** | **Duration (days), Median (95%CI)** |  | **No. (%) of responders** | **Duration (days), Median (95%CI)** | **HR (95%CI)** | **P value** |
| Return to normal body temperature | 80 (98.8%) | 1.0 (0.8~1.4) |  | 73 (98.6%) | 1.6 (1.3~1.9) | 1.37(1.00~1.89) | 0.052 |
| Gastrointestinal symptoms | 110 (93.2%) | 1.7 (1.0~1.9) |  | 94 (92.25) | 1.8 (1.4~2.0) | 1.17(0.89~1.54) | 0.259 |
| Anosmia or ageusia | 83 (88.3%) | 2.7 (1.9~4.3) |  | 81 (84.4%) | 3.6 (2.7~5.0) | 1.21(0.89~1.64) | 0.227 |
| Negative conversion of NAAT | 245 (59.8%) | 10.5 (10.0~14.0) |  | 234 (57.8%) | 14.0 (13.0~14.0) | 1.12(0.94~1.34) | 0.196 |
| Improvement of chest imaging * | 10(66.7%) | NA |  | 3(33.3%) | NA | NA | NA |

Responders denoted the patients who had a sustained improvement or resolution of the symptoms.

NA: not applicable

* Only 15 patients in LHQW group and 9 in placebo group hadthe radiologic evidence of pneumonia, which allowed for the comparison of radiologic manifestations prior to and after treatment.

**Table S7. Comparison of the treatment-emergent adverse events in the safety set**

|  | **LHQW group** | | **Placebo group** | |
| --- | --- | --- | --- | --- |
|  | **No. of patients with TEAES** | **No. of TEAES** | **No. of patients with TEAES** | **No. of TEAES** |
| **No.** | 423 |  | 425 |  |
| **Overall n(%)** | 30(7.1) | 39 | 33(7.8) | 41 |
| **Laboratory tests, n(%)** | 14(3.3) | 17 | 17(4.0) | 22 |
| Elevated interleukin-6, n(%) | 9(2.1) | 9 | 8(1.9) | 10 |
| Elevated D-dimer, n(%) | 4(0.9) | 4 | 3(0.7) | 3 |
| Elevated alanine aminotransferrase, n(%) | 1(0.2) | 1 | 3(0.7) | 3 |
| Urinary leukocytosis, n(%) | 0(0) | 0 | 1(0.2) | 1 |
| Elevated aspartate aminotransferrase, n(%) | 0(0) | 0 | 1(0.2) | 1 |
| Elevated bilirubin, n(%) | 0(0) | 0 | 1(0.2) | 1 |
| Elevated blood creatinine kinase, n(%) | 0(0) | 0 | 1(0.2) | 1 |
| Low blood glucose, n(%) | 1(0.2) | 1 | 0(0) | 0 |
| Elevated blood glucose, n(%) | 1(0.2) | 1 | 0(0) | 0 |
| Elevated blood lactate dehydrogenase, n(%) | 1(0.2) | 1 | 0(0) | 0 |
| Elevated platelet, n(%) | 0(0) | 0 | 1(0.2) | 1 |
| High blood pressure, n(%) | 0(0) | 0 | 1(0.2) | 1 |
| **Psychiatric disorders, n(%)** | 3(0.7) | 4 | 5(1.2) | 5 |
| Insomnia, n(%) | 2(0.5) | 2 | 2(0.5) | 2 |
| Sleep disorder, n(%) | 1(0.2) | 1 | 1(0.2) | 1 |
| Anxiety, n(%) | 1(0.2) | 1 | 0(0) | 0 |
| Difficulty in sleep, n(%) | 0(0) | 0 | 1(0.2) | 1 |
| Poor sleep quality, n(%) | 0(0) | 0 | 1(0.2) | 1 |
| **Cutaneous or subcutaneous disorders, n(%)** | 4(0.9) | 4 | 1(0.2) | 2 |
| Rashes, n(%) | 1(0.2) | 1 | 1(0.2) | 2 |
| Itching, n(%) | 2(0.5) | 2 | 0(0) | 0 |
| Atopic dermatitis, n(%) | 1(0.2) | 1 | 0(0) | 0 |
| **Neurological diseases, n(%)** | 2(0.5) | 2 | 2(0.5) | 2 |
| Headache, n(%) | 1(0.2) | 1 | 1(0.2) | 1 |
| Desensitized feeling, n(%) | 0(0) | 0 | 1(0.2) | 1 |
| Dizziness, n(%) | 1(0.2) | 1 | 0(0) | 0 |
| **Gastrointestinal disorders, n(%)** | 3(0.7) | 5 | 1(0.2) | 2 |
| Diarrhea, n(%) | 3(0.7) | 4 | 0(0) | 0 |
| Constipation, n(%) | 0(0) | 0 | 1(0.2) | 1 |
| Disgusting, n(%) | 1(0.2) | 1 | 0(0) | 0 |
| Gastritis, n(%) | 0(0) | 0 | 1(0.2) | 1 |
| **Vascular or lymph disorders, n(%)** | 2(0.5) | 2 | 2(0.5) | 2 |
| Hypertension, n(%) | 2(0.5) | 2 | 2(0.5) | 2 |
| **Cardiac disorders, n(%)** | 1(0.2) | 1 | 2(0.5) | 2 |
| Supraventricular tachycardia, n(%) | 0(0) | 0 | 1(0.2) | 1 |
| Ischemia, n(%) | 1(0.2) | 1 | 0(0) | 0 |
| Arrhythmia, n(%) | 0(0) | 0 | 1(0.2) | 1 |
| **Metabolic and nutritional disorders, n(%)** | 2(0.5) | 2 | 0(0) | 0 |
| Loss of appetite, n(%) | 2(0.5) | 2 | 0 (0) | 0 |
| **Nasal disorders, n (%)** | 2(0.5) | 2 | 0 (0) | 0 |
| Rhinitis, n(%) | 1(0.2) | 1 | 0(0) | 0 |
| Epistaxis, n(%) | 1(0.2) | 1 | 0(0) | 0 |
| **Infectious disorders, n(%)** | 1(0.2) | 1 | 1(0.2) | 1 |
| Herpes virus infection, n(%) | 0(0) | 0 | 1(0.2) | 1 |
| **Respiratory disorders, n(%)** | 0(0) | 0 | 1(0.2) | 1 |
| Chest pain, n(%) | 0(0) | 0 | 1(0.2) | 1 |
| **Musculoskeletal and connective tissue disorder, n(%)** | 0(0) | 0 | 1(0.2) | 1 |
| Spondylostenosis, n(%) | 0(0) | 0 | 1(0.2) | 1 |
| **Immune disorders, n(%)** | 0(0) | 0 | 1(0.2) | 1 |
| Hypersensitivity, n(%) | 0(0) | 0 | 1(0.2) | 1 |

TEAE: treatment-emergent adverse event

**Figure legends**

**Figure S1. The percentage of patients who achieved sustained improvement or resolution of the main symptoms in the per-protocol set**

Shown in the figures are the bars of the treatment group (red) and placebo group (blue)

**Figure S2. The time to the resolution of additional symptoms evaluated in the treatment group (red curve) and placebo group (blue curve) according to the full-analysis set**

Figure S2-A. Time to resolution of anosmia and ageusia;

Figure S2-B. Time to the return of body temperature to normal levels;

Figure S2-C. Time to resolution of gastrointestinal symptoms;
