# Supplementary figures and images for: Effects of Lianhuaqingwen Capsules in adults with mild-to-moderate coronavirus disease 2019: an international, multicenter, double-blind, randomized controlled trial
Source: Virol J. 2023 Nov 28;20:277. doi: 10.1186/s12985-023-02144-6 (PMC10685492; doi:10.1186/s12985-023-02144-6)

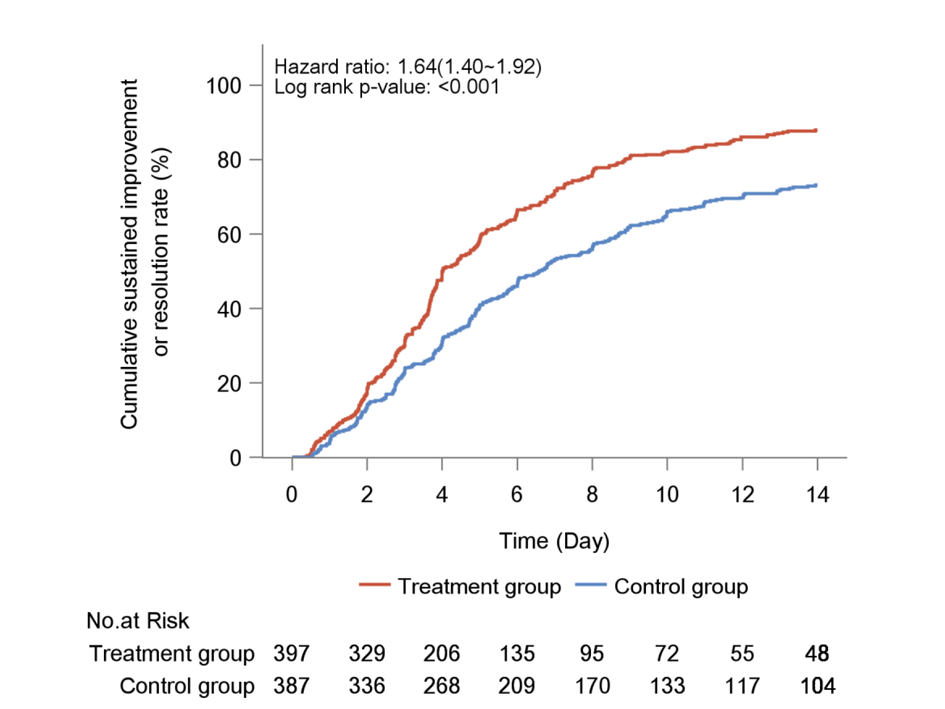

Supplement: Supplementary file 2 — Additional file 2: Figure S1. The percentage of patients who achieved sustained improvement or resolution of the main symptoms in the per-protocol set. Shown in the figures are the bars of the treatment group (red) and placebo group (blue). [file 12985_2023_2144_MOESM2_ESM.tif]

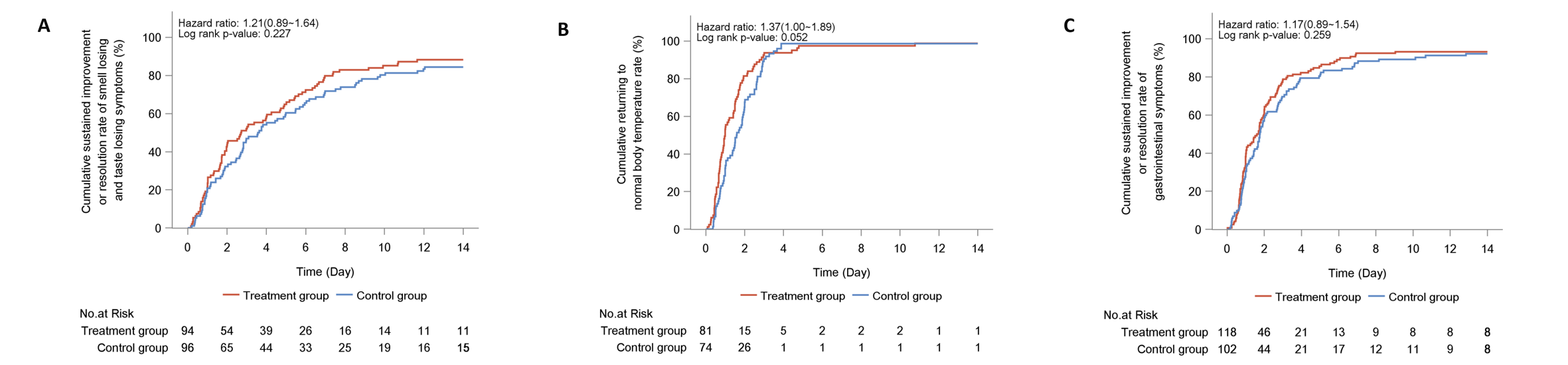

Supplement: Supplementary file 3 — Additional file 3: Figure S2. The time to the resolution of additional symptoms evaluated in the treatment group (red curve) and placebo group (blue curve) according to the full-analysis set. A Time to resolution of anosmia and ageusia; B Time to the return of body temperature to normal levels; C Time to resolution of gastrointestinal symptoms. [file 12985_2023_2144_MOESM3_ESM.tif]
